# Assessment of Patient Attribution to Care From Medical Oncologists, Surgeons, or Radiation Oncologists After Newly Diagnosed Cancer

**DOI:** 10.1001/jamanetworkopen.2021.8055

**Published:** 2021-05-10

**Authors:** Suhas Gondi, Alexi A. Wright, Mary Beth Landrum, Laurie Meneades, Jose Zubizarreta, Michael E. Chernew, Nancy L. Keating

**Affiliations:** 1 Department of Health Care Policy, Harvard Medical School, Boston, Massachusetts; 2 Division of Population Sciences, Dana-Farber Cancer Institute, Boston, Massachusetts; 3 Division of General Internal Medicine, Brigham and Women’s Hospital, Boston, Massachusetts

## Abstract

This cohort study uses data from the Surveillance, Epidemiology, and End Results–Medicare Linked Database to assess the attribution of patients with newly diagnosed lung, breast, colorectal, or prostate cancer to care from multidisciplinary specialists—medical oncologists, surgeons, or radiation oncologists—within 6 months after diagnosis.

## Introduction

As payers implement value-based payments for oncology care, assignment of patients to physician practices is increasingly important to accurately assess quality and reimburse clinicians accordingly. Yet, patient attribution remains a challenge.^1^ Most claims-based attribution algorithms assign patients to practices based on the plurality of primary care visits. However, clinician attribution for specialty care is complex. The challenges of attribution are particularly salient in oncology because cancer care is often multidisciplinary—involving medical oncologists, surgeons, and radiation oncologists—rendering it difficult to discern which practice should be held accountable.^2^ We sought to identify practices treating Medicare beneficiaries with a new diagnosis of cancer to inform potential attribution algorithms based on care received in the 6 months after diagnosis.

## Methods

We used data from the Surveillance, Epidemiology, and End Results (SEER)–Medicare Linked Database (2010-2016) for analyses. The SEER program collects data from population-based cancer registries^3^; these data are linked with Medicare administrative data.^4^ We identified traditional (fee-for-service) Medicare beneficiaries 65 years or older who had received a new diagnosis of invasive breast, colorectal, lung, or prostate cancer between January 1, 2011, and December 31, 2015 (eTable 1 in eAppendix 1 in the Supplement), and examined claims through 6 months after diagnosis. The Harvard Medical School Committee on Human Studies approved the study. A waiver of patient informed consent was obtained because patient identifiers are not included in the SEER-Medicare data. We followed the Strengthening the Reporting of Observational Studies in Epidemiology (STROBE) reporting guideline for cohort studies.

We attributed patients to practices based on outpatient evaluation and management (E&M) claims with a cancer diagnosis in the 6 months after diagnosis (eTables 2 through 7 in eAppendixes 2 through 5 in the Supplement). We attributed patients to the practice with the most E&M visits and to medical oncology, surgery, or radiation oncology practices. We also assessed whether inclusion of inpatient E&M claims improved attribution rates. Finally, we described the proportion of patients who visited more than 1 practice of each type. This analysis was performed between August 1, 2019, and November 30, 2020. No statistical testing was conducted for this descriptive study.

## Results

The 301 327 patients with newly diagnosed lung, breast, colorectal, or prostate cancer had a mean (SD) age of 75.1 (7.3) years, 149 485 (49.6%) were male, and 241 232 (80.0%) were White patients (Table 1). Only 77.9% of patients with colorectal cancer and 74.1% of patients with lung cancer were attributed to a practice based on outpatient E&M visits (Table 2). These numbers increased to 90.4% and 87.6%, respectively, when inpatient E&M claims were included. Most patients with breast cancer (73.2%), colorectal cancer (61.6%), and lung cancer (65.3%) had visits with a medical oncologist, but only 11.3% of patients with prostate cancer did.

**Table 1.** Characteristics of the Study Population of 301 327 Patients

Characteristic	No. (%)^a^
Age, mean (SD), y	75.1 (7.3)
Cancer type	
Breast	78 736 (26.1)
Colorectal	51 385 (17.0)
Lung	95 635 (31.9)
Prostate	75 571 (25.0)
Sex	
Male	149 485 (49.6)
Female	151 842 (50.4)
Race/ethnicity	
White	241 232 (80.0)
Black	26 650 (8.8)
Hispanic	15 991 (5.3)
Asian/Pacific Islander	13 537 (4.5)
Other^b^	3917 (1.3)
Marital status	
Married	155 337 (52.0)
Single/divorced/separated/widowed	122 485 (40.6)
Unknown	23 505 (7.8)
Year of diagnosis	
2011	64 185 (21.3)
2012	61 478 (20.4)
2013	59 317 (19.7)
2014	57 960 (19.2)
2015	58 387 (19.4)
Charlson Comorbidity Index	
0	116 382 (38.6)
1	73 049 (24.4)
2	43 857 (14.2)
≥3	68 039 (22.6)
SEER registry sites	
San Francisco-Oakland/San Jose-Monterey	17 804 (5.9)
Connecticut	16 249 (5.4)
Detroit	18 429 (6.1)
Hawaii	3181 (1.0)
Iowa	17 778 (5.9)
New Mexico	5877 (2.0)
Seattle-Puget Sound	18 135 (6.0)
Utah	5710 (1.9
Los Angeles	17 340 (5.8)
Greater California	55 755 (18.5)
Kentucky	23 668 (7.9)
Louisiana	19 974 (6.7)
New Jersey	45 134 (15.0)
Georgia	36 293 (12.0)
Cancer-directed surgery within 6 mo	
No	165 129 (54.8)
Yes	136 198 (45.2)
Chemotherapy within 6 mo	
No	180 885 (60.0)
Yes	120 442 (40.0)
Radiation within 6 mo	
No	222 558 (73.8)
Yes	78 769 (26.2)

^a^ Percentages may not total 100 because of rounding.

^b^ Other racial/ethnic groups include 1119 American Indian/Alaska Native patients and 2798 patients with unknown race/ethnicity.

**Table 2.** Attribution of Patients With Newly Diagnosed Cancer to Medical Oncology, Surgery, and Radiation Oncology Practices

Practice assignment	Type of cancer, No. (%) or No./total No. (%)^a^			
	Breast (n = 78 736)	Colorectal (n = 51 385)	Lung (n = 95 635)	Prostate (n = 75 571)
**Proportion of patients attributed to practices based on visits in the 6 mo after diagnosis**				
Assigned to any practice type				
Based on outpatient visits	72 291 (91.8)	40 054 (77.9)	70 854 (74.1)	67 390 (89.2)
Based on outpatient and inpatient visits	73 258 (93.0)	46 439 (90.4)	83 797 (87.6)	68 201 (90.2)
Based on outpatient visits, stratified by cancer stage				
Stage I	33 036/33 942 (97.3)	9384/12 225 (76.8)	19 129/23 132 (82.7)	399/681 (58.6)
Stage II	19 684/20 440 (96.3)	11 734/14 316 (82.0)	3478/3996 (87.0)	51 946/57 896 (89.7)
Stage III	5448/5698 (95.6)	11 011/12 810 (86.0)	17 128/22 326 (76.7)	4840/5122 (94.5)
Stage IV	3304/4138 (79.8)	7212/10 482 (68.8)	29 033/42 541 (68.2)	6975/8288 (84.2)
Assigned to any medical oncology practice^b^				
Based on outpatient visits	56 884 (72.2)	27 857 (54.2)	53 635 (56.1)	8010 (10.6)
Based on outpatient and inpatient visits	57 631 (73.2)	31 647 (61.6)	62 458 (65.3)	8548 (11.3)
Based on outpatient visits by cancer stage				
Stage I	27 453/33 942 (80.9)	4163/12 225 (34.1)	10 468/23 132 (45.3)	8/681 (1.2)
Stage II	16 888/20 440 (82.6)	7969/14 316 (55.7)	2870/3996 (71.8)	3658/57 896 (6.3)
Stage III	4817/5698 (84.5)	9238/12 810 (72.1)	14 361/22 326 (64.3)	491/5122 (9.6)
Stage IV	2939/4138 (71.0)	6114/10 482 (58.3)	24 651/42 541 (57.9)	3640/8288 (43.9)
Assigned to any surgical practice				
Based on surgery claim	65 849 (83.6)	36 733 (71.5)	16 939 (17.7)	13 974 (18.5)
Based on surgery claim or visit to a surgeon	73 599 (93.5)	42 993 (83.7)	35 527 (37.1)	67 722 (89.6)
Stratified by cancer stage				
Stage I	23 928/33 942 (97.0)	10 375/12 225 (84.9)	15 296/23 132 (66.1)	552/681 (81.1)
Stage II	19 482/20 440 (95.3)	13 497/14 316 (94.3)	2550/3996 (63.8)	52 803/57 896 (91.2)
Stage III	5301/5698 (93.0)	12 051/12 810 (94.1)	7818/22 326 (35.0)	4846/5122 (94.6)
Stage IV	2208/4138 (53.4)	6166/10 482 (58.8)	8506/42 541 (20.0)	6200/8288 (74.8)
Assigned to any radiation oncology practice				
Based on radiation claim	9432 (12.0)	1647 (3.2)	8404 (8.8)	11 503 (15.2)
Based on radiation claim or visit to a radiation oncologist	42 221 (53.6)	6083 (11.8)	34 086 (35.6)	34 208 (45.3)
Stratified by cancer stage				
Stage I	21 647/33 942 (63.8)	1108/12 225 (9.1)	7998/23 132 (34.6)	32/681 (4.7)
Stage II	9769/20 440 (47.8)	1823/14 316 (12.7)	1680/3996 (42.0)	28 480/57 896 (49.2)
Stage III	2250/5698 (39.5)	2053/12 810 (16.0)	9771/22 326 (43.8)	2002/5122 (39.1)
Stage IV	1011/4138 (24.4)	936/10 482 (8.9)	13 799/42 541 (32.4)	2240/8288 (27.0)
**Proportion of patients with cancer-related visits to >1 practice in the 6 mo after diagnosis**				
Outpatients with visits to >1 practice of any type	56 223/78 736 (71.4)	26 084/51 385 (50.8)	47 774/95 635 (50.0)	42 812/75 571 (56.7)
Outpatients with any visits to medical oncology practice^b^				
Outpatients with visits to >1 medical oncology practice	4489/56 684 (7.9)	2285/27 857 (8.2)	5492/53 635 (10.2)	599/8010 (7.5)
Outpatients with only 1 visit to medical oncology practice	12 443/56 684 (21.9)	6467/27 857 (23.2)	11 181/53 635 (20.8)	3218/8010 (40.2)
Outpatients with surgery visits or surgery				
Outpatients with surgical visits or surgical procedures from >1 surgery practice	15 184/73 599 (20.6)	9540/42 993 (22.2)	5522/35 527 (15.5)	15 338/67 722 (22.6)
Outpatients with ≥1 radiation oncology visit or radiation				
Outpatients with visits to or radiation therapy from > l radiation oncology practice	2313/42 221 (5.5)	268/6083 (4.4)	1925/34 086 (5.6)	3593/34 208 (10.5)

^a^ Percentages may not total 100 because of rounding.

^b^ A visit to a medical oncology practice was defined as a claim with a specialty code of medical oncology, hematology/oncology, hematology, or gynecologic oncology.

Attribution based on all cancer-related visits and medical oncology visits varied by cancer type and stage (Table 2). For example, only 34.1% of patients with stage I colorectal cancer had a medical oncology visit within 6 months of diagnosis vs 72.1% of those with stage III cancers. Most patients had cancer-related outpatient visits to multiple practices. For example, 71.4% of patients with breast cancer, 50.8% with colorectal cancer, 50.0% with lung cancer, and 56.7% with prostate cancer had visits to multiple practices (Table 2). Across cancer types, 7.5% to 10.2% of patients had outpatient visits with more than 1 medical oncology practice.

## Discussion

Our analysis reveals the challenges of attribution of patients with newly diagnosed cancer that should be addressed for accurate quality measurement and emerging value-based payments in oncology.^5,6^ First, many patients with newly diagnosed lung or colorectal cancer were not attributed to a practice based on outpatient E&M claims alone. Efforts seeking to characterize practice-level quality for patients who may receive only inpatient care (eg, early-stage colon cancer, metastatic lung cancer) should include inpatient E&M or procedure claims.

Second, attribution varied substantially by cancer type and stage, underscoring the importance of considering the clinical context of the care being delivered. For instance, approximately a quarter to a third of patients with breast, colorectal, and lung cancers and 88% of patients with prostate cancer had no medical oncologist visits. These patterns are consistent with medical indications (ie, many patients with early-stage disease do not require chemotherapy) and clinical norms (eg, patients with prostate cancer are primarily treated by urologists). Attribution algorithms ideally would consider cancer stage and tumor characteristics. Unfortunately, such variables are not available in claims data, creating a need to leverage other data sources to collect inputs to attribution algorithms.

Third, many patients have cancer-related visits to multiple practices. The payment methodology and application of quality metrics should be tailored to the type of clinician and type of care delivered by a practice (eg, surgery, systemic therapy, radiation). Some patients have multiple visits to the same type of clinician at different practices (8% to 11% of those who saw a medical oncologist had visits to >1 practice). In such cases, it is challenging to determine the practice accountable for care.

Our study is limited by its focus on traditional Medicare beneficiaries living in SEER areas. The generalizability of our findings to commercially insured populations or individuals in other areas requires further study.

## Notes

Supplement. **eAppendix 1.** Study Population **eTable 1.** CONSORT Diagram for Study Population **eAppendix 2.** Methods for Identifying Outpatient and Inpatient Visits With Physicians and Attribution to Practices **eTable 2.** Diagnosis Codes for Breast, Colorectal, Lung, and Prostate Cancer **eAppendix 3.** Attribution to Medical Oncology Practices **eAppendix 4.** Attribution to Surgery Practices **eTable 3.** Lung Cancer Surgery Codes **eTable 4.** Colorectal Cancer Surgery Codes **eTable 5.** Breast Cancer Surgery Codes **eTable 6.** Prostate Cancer Surgery Codes **eAppendix 5.** Attribution to Radiation Oncology Practices **eTable 7.** Radiation Delivery Codes

## References

[1] Mehrotra A, Burstin H, Raphael C. Raising the bar in attribution. Ann Intern Med. 2017;167(6):434-435. doi:10.7326/M17-0655 28847009

[2] Gondi S, Wright AA, Landrum MB, Zubizarreta J, Chernew ME, Keating NL. Multimodality cancer care and implications for episode-based payments in cancer. Am J Manag Care. 2019;25(11):537-538. 31747230

[3] Warren JL, Klabunde CN, Schrag D, Bach PB, Riley GF. Overview of the SEER-Medicare data: content, research applications, and generalizability to the United States elderly population. Med Care. 2002;40(8)(suppl):IV-3-IV-18. doi:10.1097/00005650-200208001-00002 12187163

[4] Potosky AL, Riley GF, Lubitz JD, Mentnech RM, Kessler LG. Potential for cancer related health services research using a linked Medicare-tumor registry database. Med Care. 1993;31(8):732-748. doi:10.1097/00005650-199308000-00006 8336512

[5] Howard DH, Torres MA. Alternative payment for radiation oncology. JAMA. 2019;322(19):1859-1860. doi:10.1001/jama.2019.15888 31596451

[6] Aviki EM, Schleicher SM, Mullangi S, Matsoukas K, Korenstein D. Alternative payment and care-delivery models in oncology: a systematic review. Cancer. 2018;124(16):3293-3306. doi:10.1002/cncr.31367 30141837 PMC6110102

