# Supplementary material for: Assessment of Patient Attribution to Care From Medical Oncologists, Surgeons, or Radiation Oncologists After Newly Diagnosed Cancer
Source: JAMA Netw Open. 2021 May 10;4(5):e218055. doi: 10.1001/jamanetworkopen.2021.8055 (PMC8111479; doi:10.1001/jamanetworkopen.2021.8055)
Supplement: Supplement. — eAppendix 1. Study Population eTable 1. CONSORT Diagram for Study Population eAppendix 2. Methods for Identifying Outpatient and Inpatient Visits With Physicians and Attribution to Practices eTable 2. Diagnosis Codes for Breast, Colorectal, Lung, and Prostate Cancer eAppendix 3. Attribution to Medical Oncology Practices eAppendix 4. Attribution to Surgery Practices eTable 3. Lung Cancer Surgery Codes eTable 4. Colorectal Cancer Surgery Codes eTable 5. Breast Cancer Surgery Codes eTable 6. Prostate Cancer Surgery Codes eAppendix 5. Attribution to Radiation Oncology Practices eTable 7. Radiation Delivery Codes [file jamanetwopen-e218055-s001.pdf]

SUPPLEMENTARY ONLINE CONTENT

Gondi S, Wright AA, Landrum MB, et al. Assessment of patient attribution to care from medical oncologists, surgeons, or radiation oncologists after a newly diagnosed cancer.
JAMA Netw Open. 2021;4(5):e218055. doi:10.1001/jamanetworkopen.2021.8055

eAppendix 1. Study Population

eTable 1. CONSORT Diagram for Study Population

eAppendix 2. Methods for Identifying Outpatient and Inpatient Visits With Physicians and Attribution to Practices

eTable 2. Diagnosis Codes for Breast, Colorectal, Lung, and Prostate Cancer

eAppendix 3. Attribution to Medical Oncology Practices

eAppendix 4. Attribution to Surgery Practices

eTable 3. Lung Cancer Surgery Codes

eTable 4. Colorectal Cancer Surgery Codes

eTable 5. Breast Cancer Surgery Codes

eTable 6. Prostate Cancer Surgery Codes

eAppendix 5. Attribution to Radiation Oncology Practices

eTable 7. Radiation Delivery Codes

This supplementary material has been provided by the authors to give readers additional information about their work.

© 2021 Gondi S et al. *JAMA Network Open.*

eAppendix 1. Study Population

eTable 1. CONSORT Diagram for Study Population

**Inclusion Criteria**	**Colorectal**	**Lung**	**Breast**	**Prostate**	**Total**
Patients diagnosed with cancer in 2011-2015	125,626	194,750	197,504	194,536	712,416
Patients with non-missing month of diagnosis	125,041	194,029	196,767	192,815	708,652
Patients not identified by autopsy or death certificate	123,586	189,016	195,674	191,010	699,286
Patients with cancer stage information	117,708	181,220	193,162	182,411	674,501
Patients who are not stage 0*	110,061	180,769	---	182,411	473,241
Patients continuously enrolled in Parts A and B of FFS Medicare for 6 months after diagnosis or until death	55,773	105,071	87,237	81,432	329,513
Patients without end-stage renal disease	55,445	104,601	86,841	80,984	327,871
Patients who are aged 65+	51,385	95,635	79,617	75,571	302,208
Patients who are female (breast)	---	---	78,736	---	78,736

*The breast cancer cohort did not exclude stage 0 patients.

© 2021 Gondi S et al. *JAMA Network Open.*

eAppendix 2. Methods for Identifying Outpatient and Inpatient Visits With Physicians and Attribution to Practices

We attributed patients to practices in the 6 months after diagnosis based on evaluation and management (E&M) claims for outpatient physician visits with a cancer diagnosis. Outpatient visits were identified based on Current Procedural Terminology (CPT) codes 99201-99215, 99241-99245. We required the claim to include the specific cancer diagnosis:

eTable 2. Diagnosis Codes for Breast, Colorectal, Lung, and Prostate Cancer

**Cancer**	**Diagnosis Codes**
Breast	Icd-9: 174xx,175xx
	Icd-10: C50011,C50012,C50019,C50111,C50112,C50119,C50211,C50212,C50219,
	C50311,C50312,C50319,C50411,C50412,C50419,C50511,C50512,C50519,
	C50611,C50612,C50619,C50811,C50812,C50819,C50911,C50912,C50919
	C50021,C50022,C50029,C50121,C50122,C50129,C50221,C50222,C50229,
	C50321,C50322,C50329,C50421,C50422,C50429,C50521,C50522,C50529,
	C50621,C50622,C50629,C50821,C50822,C50829,C50921,C50922,C50929
Colorectal	Icd-9: 153xx 154xx
	Icd-10: C180-C20x
Lung	Icd-9: 162xx
	Icd-10: C3400 ,C3401 ,C3402 ,C3410 ,C3411 ,C3412 ,C342 ,C3430 ,C3431 ,C3432 , C3480 ,C3481 ,C3482 ,C3490 ,C3491 ,C3492
Prostate	Icd-9: 185xx
	Icd-10: C61xx

Patients were assigned to the practice (based on Tax Identification Number) with the most E&M cancer-related visits; if there was a tie, we assigned patients to the practice with the most recent visit. We included all practices who were providing cancer-related care (including care by surgeons, radiation oncologists, medical oncologists, urologists, internists, etc.).

In addition, we tested the inclusion of inpatient E&M claims with a cancer diagnosis. Inpatient physician visits were identified based on CPT codes 99217-99226, 99231-99236, 99238-99239, 99251-99255, 99281-99285.

The following sections describe attribution to medical oncology, surgery, and radiation oncology practices specifically. Note that there are several possibilities for why a patient may not be attributed with our methods, including they did not seek treatment, were diagnosed by a primary care physician but did not seek follow-up, only saw non-oncologists for cancer care, or saw oncologists who billed under a non-oncology specialty code like “internal medicine.”

© 2021 Gondi S et al. *JAMA Network Open.*

eAppendix 3. Attribution to Medical Oncology Practices

Identification of visits to a “medical oncology practice” was based on E&M visit code with a CMS specialty code of medical oncology (83), hematology/oncology (90), hematology (82), or gynecologic oncology (98). These are the specialties that typically provide chemotherapy to patients.

© 2021 Gondi S et al. *JAMA Network Open.*

eAppendix 4. Attribution to Surgery Practices

For attribution to surgical practices, we identified patients with claims in the carrier file for surgeries of interest during the month of diagnosis through 6 months after diagnosis and identified the TIN on the surgery claim. If patients had more than one claim for a surgery, we selected the claim for the surgery with the largest allowed amount. If patients had two of the same surgical procedure, we selected the claim for the most recent surgery. Surgeries included are listed below by cancer type. Note that for TIN assignment, we focused on the carrier claims (and associated HCPCS codes) so that we could attribute to a TIN.

If patients did not have a claim for a cancer directed surgery, we identified if they had an E&M visit to a surgery practice using CMS specialty codes of general surgery (02) or surgical oncology (91) (for colorectal cancer, we also included colorectal surgery (28); for lung cancer, we also included thoracic surgery (78) and cardiac surgery (33); for prostate cancer we also included urology (34).

Procedure codes by cancer type

eTable 3. Lung Cancer Surgery Codes

**Procedure - Pneumonectomy**
**CPT**
32440
32442
32445
**ICD-9**
32.50
32.59
**Procedure - Lobectomy**
**CPT**
32480
32482
32484
32486
32663
**ICD-9**
32.41
32.49
32.6
**Procedure - Wedge or Segmental Resection**
**CPT**
32500
32505
32506
32507
32657

© 2021 Gondi S et al. *JAMA Network Open.*

32666
32667
32668
**ICD-9**
32.30
32.39

© 2021 Gondi S et al. *JAMA Network Open.*

eTable 4. Colorectal Cancer Surgery Codes

CPT Codes	Definition	Resection Type
44140	Colectomy, partial; with anastomosis	Colon
44141	Colectomy, partial; with skin level cecostomy or colostomy	Colon
44143	Colectomy, partial; with end colostomy and closure of distal segment (Hartmann type procedure)	Colon
44144	Colectomy, partial; with resection, with colostomy or ileostomy and creation of mucofistula	Colon
44145	Colectomy, partial; with coloproctostomy (low pelvic anastomosis)	Colon
44146	Colectomy, partial; with coloproctostomy (low pelvic anastomosis), with colostomy	Colon
44147	Colectomy, partial; abdominal and transanal approach	Colon
44150	Colectomy, total, abdominal, without proctectomy; with ileostomy or ileoproctostomy	Colon
44151	Colectomy, total, abdominal, without proctectomy; with continent ileostomy	Colon
44155	Colectomy, total, abdominal, with proctectomy; with ileostomy	colon+rectum
44156	Colectomy, total, abdominal, with proctectomy; with continent ileostomy	colon+rectum
44157	Colectomy, total, abdominal, with proctectomy; with ileoanal anastomosis, includes loop ileostomy, and rectal mucosectomy, when performed	colon+rectum
44158	Colectomy, total, abdominal, with proctectomy; with ileoanal anastomosis, creation of ileal reservoir (S or J), includes loop ileostomy, and rectal mucosectomy, when performed	colon+rectum
44160	Colectomy, partial, with removal of terminal ileum with ileocolostomy	Colon
44204	Laparoscopy, surgical; colectomy, partial, with anastomosis	Colon
44205	Laparoscopy, surgical; colectomy, partial, with removal of terminal ileum with ileocolostomy	Colon
44206	Laparoscopy, surgical; colectomy, partial, with end colostomy and closure of distal segment (Hartmann type procedure)	Colon
44207	Laparoscopy, surgical; colectomy, partial, with anastomosis, with coloproctostomy (low pelvic anastomosis)	Colon
44208	Laparoscopy, surgical; colectomy, partial, with anastomosis, with coloproctostomy (low pelvic anastomosis) with colostomy	Colon
44210	Laparoscopy, surgical; colectomy, total, abdominal, without proctectomy, with ileostomy or ileoproctostomy	Colon
44211	Laparoscopy, surgical; colectomy, total, abdominal, with proctectomy, with ileoanal anastomosis, creation of ileal reservoir (S or J), with loop ileostomy, includes rectal mucosectomy, when performed	colon+rectum
44212	Laparoscopy, surgical; colectomy, total, abdominal, with proctectomy, with ileostomy	colon+rectum
44213	Laparoscopy, surgical, mobilization (take-down) of splenic flexure performed in conjunction with partial colectomy (List separately in addition to primary procedure)	Colon
45110	Proctectomy; complete, combined abdominoperineal, with colostomy	Rectal
45111	Proctectomy; partial resection of rectum, transabdominal approach	Rectal

© 2021 Gondi S et al. *JAMA Network Open.*

45112	Proctectomy, combined abdominoperineal, pull-through procedure (eg, colo-anal anastomosis)	Rectal
45113	Proctectomy, partial, with rectal mucosectomy, ileoanal anastomosis, creation of ileal reservoir (S or J), with or without loop ileostomy	Rectal
45114	Proctectomy, partial, with anastomosis; abdominal and transsacral approach	Rectal
45116	Proctectomy, partial, with anastomosis; transsacral approach only (Kraske type)	Rectal
45119	Proctectomy, combined abdominoperineal pull-through procedure (eg, colo-anal anastomosis), with creation of colonic reservoir (eg, J-pouch), with diverting enterostomy when performed	Rectal
45120	Proctectomy, complete (for congenital megacolon), abdominal and perineal approach; with pull-through procedure and anastomosis (eg, Swenson, Duhamel, or Soave type operation)	Rectal
45121	Proctectomy, complete (for congenital megacolon), abdominal and perineal approach; with subtotal or total colectomy, with multiple biopsies	colon+rectum
45123	Proctectomy, partial, without anastomosis, perineal approach	Rectal
45126	Pelvic exenteration for colorectal malignancy, with proctectomy (with or without colostomy), with removal of bladder and ureteral transplantations, and/or hysterectomy, or cervicectomy, with or without removal of tube(s), with or without removal of ovary(s), or any combination thereof	Rectal

Note: did not add/include: 44227: Laparoscopy, surgical, closure of enterostomy, large or small intestine, with resection and anastomosis; 44238: Unlisted laparoscopy procedure, intestine (except rectum)

© 2021 Gondi S et al. *JAMA Network Open.*

eTable 5. Breast Cancer Surgery Codes

**Lumpectomy**	
**CPT Codes**	**Description**
19120	Excision of cyst, fibroadenoma, or other benign or malignant tumor, aberrant breast tissue, duct lesion, nipple or areolar lesion (except 19300), open, male or female, 1 or more lesions
19125	Excision of breast lesion identified by preoperative placement of radiological marker, open; single lesion
19126	Excision of breast lesion identified by preoperative placement of radiological marker, open; each additional lesion separately identified by a preoperative radiological marker (List separately in addition to code for primary procedure)
19160	Mastectomy, partial (eg, lumpectomy, tylectomy, quadrantectomy, segmentectomy);
19162	Mastectomy, partial (eg, lumpectomy, tylectomy, quadrantectomy, segmentectomy); with axillary lymphadenectomy
19301	Mastectomy, partial (eg, lumpectomy, tylectomy, quadrantectomy, segmentectomy);
19302	Mastectomy, partial (eg, lumpectomy, tylectomy, quadrantectomy, segmentectomy); with axillary lymphadenectomy
**ICD-9 Codes**	**Description**
85.21	Local excision of lesion of breast
85.22	Resection of quadrant of breast
85.23	Subtotal mastectomy
**Mastectomy**	
**CPT Codes**	**Description**
19180	Mastectomy, simple, complete
19182	Mastectomy, subcutaneous
19200	Mastectomy, radical, including pectoral muscles, axillary lymph nodes
19220	Mastectomy, radical, including pectoral muscles, axillary and internal mammary lymph nodes (Urban type operation)
19240	Mastectomy, modified radical, including axillary lymph nodes, with or without pectoralis minor muscle, but excluding pectoralis major muscle
19303	Mastectomy, simple, complete
19304	Mastectomy, subcutaneous
19305	Mastectomy, radical, including pectoral muscles, axillary lymph nodes
19306	Mastectomy, radical, including pectoral muscles, axillary and internal mammary lymph nodes (Urban type operation)
19307	Mastectomy, modified radical, including axillary lymph nodes, with or without pectoralis minor muscle, but excluding pectoralis major muscle
**ICD-9 Codes**	**Description**
85.41	Unilateral simple mastectomy
85.42	Bilateral simple mastectomy
85.43	Unilateral extended simple mastectomy
85.44	Bilateral extended simple mastectomy

© 2021 Gondi S et al. *JAMA Network Open.*

85.45	Unilateral radical mastectomy
85.46	Bilateral radical mastectomy
85.47	Unilateral extended radical mastectomy
85.48	Bilateral extended radical mastectomy

© 2021 Gondi S et al. *JAMA Network Open.*

eTable 6. Prostate Cancer Surgery Codes

**Prostatectomy**	
**CPT**	**Definition**
55810	Prostatectomy, perineal radical;
55812	Prostatectomy, perineal radical; with lymph node biopsy(s) (limited pelvic lymphadenectomy)
55815	Prostatectomy, perineal radical; with bilateral pelvic lymphadenectomy, including external iliac, hypogastric and obturator nodes
55840	Prostatectomy, retropubic radical, with or without nerve sparing;
55842	Prostatectomy, retropubic radical, with or without nerve sparing; with lymph node biopsy(s) (limited pelvic lymphadenectomy)
55845	Prostatectomy, retropubic radical, with or without nerve sparing; with bilateral pelvic lymphadenectomy, including external iliac, hypogastric, and obturator nodes
55866	Laparoscopy, surgical prostatectomy, retropubic radical, including nerve sparing, includes robotic assistance, when performed
**ICD-9 Procedure**	
60.5	Radical prostatectomy

© 2021 Gondi S et al. *JAMA Network Open.*

eAppendix 5. Attribution to Radiation Oncology Practices

For attribution to radiation oncology practices, we identified patients with claims in the carrier file for receipt of radiation therapy during the month of diagnosis through 6 months after diagnosis and identified the TIN on the first. radiation claim. Radiation codes included are listed below.

If patients did not have a claim for radiation delivery, we identified if they had an E&M visit to a radiation oncologist using CMS specialty codes of for radiation oncology (92).

eTable 7. Radiation Delivery Codes

Code Type	CPT	Definition
CPT	77402	Radiation treatment delivery,=>1 MeV; simple
CPT	77403	Radiation treatment delivery, single treatment area, single port or parallel opposed ports, simple blocks or no blocks; 6-10 MeV
CPT	77404	Radiation treatment delivery, single treatment area, single port or parallel opposed ports, simple blocks or no blocks; 11-19 MeV
CPT	77406	Radiation treatment delivery, single treatment area, single port or parallel opposed ports, simple blocks or no blocks; 20 MeV or greater
CPT	77407	Radiation treatment delivery, =>1 MeV; intermediate
CPT	77408	Radiation treatment delivery, 2 separate treatment areas, 3 or more ports on a single treatment area, use of multiple blocks; 6-10 MeV
CPT	77409	Radiation treatment delivery, 2 separate treatment areas, 3 or more ports on a single treatment area, use of multiple blocks; 11-19 MeV
CPT	77411	Radiation treatment delivery, 2 separate treatment areas, 3 or more ports on a single treatment area, use of multiple blocks; 20 MeV or greater
CPT	77412	Radiation treatment delivery, =>1 MeV; complex
CPT	77413	Radiation treatment delivery, 3 or more separate treatment areas, custom blocking, tangential ports, wedges, rotational beam, compensators, electron beam; 6-10 MeV
CPT	77414	Radiation treatment delivery, 3 or more separate treatment areas, custom blocking, tangential ports, wedges, rotational beam, compensators, electron beam; 11-19 MeV
CPT	77416	Radiation treatment delivery, 3 or more separate treatment areas, custom blocking, tangential ports, wedges, rotational beam, compensators, electron beam; 20 MeV or greater
CPT	77418	Intensity modulated treatment delivery, single or multiple fields/arcs, via narrow spatially and temporally modulated beams, binary, dynamic MLC, per treatment session
CPT	77522	Proton treatment delivery; simple, with compensation
CPT	77523	Proton treatment delivery; intermediate
CPT	77525	Proton treatment delivery; complex
CPT	0073T	Compensator-based beam modulation treatment delivery of inverse planned treatment using 3 or more high resolution (milled or cast) compensator convergent beam modulated fields, per treatment session
CPT	77385	Intensity modulated radiation treatment delivery (IMRT), includes guidance and tracking, when performed; simple

© 2021 Gondi S et al. *JAMA Network Open.*

HCPCS	G6003	Radiation treatment delivery, single treatment area, single port or parallel opposed ports, simple blocks or no blocks: up to 5 mev
HCPCS	G6004	Radiation treatment delivery, single treatment area, single port or parallel opposed ports, simple blocks or no blocks: 6-10 mev
HCPCS	G6005	Radiation treatment delivery, single treatment area, single port or parallel opposed ports, simple blocks or no blocks: 11-19 mev
HCPCS	G6006	Radiation treatment delivery, single treatment area, single port or parallel opposed ports, simple blocks or no blocks: 20 mev or greater
HCPCS	G6007	Radiation treatment delivery, 2 separate treatment areas, 3 or more ports on a single treatment area, use of multiple blocks: up to 5 mev
HCPCS	G6008	Radiation treatment delivery, 2 separate treatment areas, 3 or more ports on a single treatment area, use of multiple blocks: 6-10 mev
HCPCS	G6009	Radiation treatment delivery, 2 separate treatment areas, 3 or more ports on a single treatment area, use of multiple blocks: 11-19 mev
HCPCS	G6010	Radiation treatment delivery, 2 separate treatment areas, 3 or more ports on a single treatment area, use of multiple blocks: 20 mev or greater
HCPCS	G6011	Radiation treatment delivery, 3 or more separate treatment areas, custom blocking, tangential ports, wedges, rotational beam, compensators, electron beam; up to 5 mev
HCPCS	G6012	Radiation treatment delivery, 3 or more separate treatment areas, custom blocking, tangential ports, wedges, rotational beam, compensators, electron beam; 6-10 mev
HCPCS	G6013	Radiation treatment delivery, 3 or more separate treatment areas, custom blocking, tangential ports, wedges, rotational beam, compensators, electron beam; 11-19 mev
HCPCS	G6014	Radiation treatment delivery, 3 or more separate treatment areas, custom blocking, tangential ports, wedges, rotational beam, compensators, electron beam; 20 mev or greater
HCPCS	G6015	Intensity modulated treatment delivery, single or multiple fields/arcs, via narrow spatially and temporally modulated beams, binary, dynamic MLC, per treatment session
HCPCS	G6016	Compensator-based beam modulation treatment delivery of inverse planned treatment using 3 or more high resolution (milled or cast) compensator, convergent beam modulated fields, per treatment session

© 2021 Gondi S et al. *JAMA Network Open.*
